# Supplementary material for: Transcription Factor MdbHLH093 Enhances Powdery Mildew Resistance by Promoting Salicylic Acid Signaling and Hydrogen Peroxide Accumulation
Source: Int J Mol Sci. 2023 May 28;24(11):9390. doi: 10.3390/ijms24119390 (PMC10253276; doi:10.3390/ijms24119390)
Supplement: Supplementary file 1 [file ijms-24-09390-s001.zip › ijms-2315861-supplementary.pdf]

Supplementary Table S1. Related primers sequence designed in this experiment.

Name	Accession number	Forward (F) and Reverse (R) primer sequence (5’-3’)	
GC-*MdbHLH093*	MDP0000644807	F	ATGGAGTTGAATGAACATGGTTTCT
		R	GAAGCATCTTCCTCCATATCCTGCA
MdbHLH093-GFP-*KpnI*		F	acgggggacgagctcggtaccATGGAGTTGAATGAACATGGTTTCT
MdbHLH093-GFP-*BamHI*		R	ggtgtcgactctagaggatccGAAGCATCTTCCTCCATATCCTGCA
pDONR222-RNAi-*MdbHLH093*		F	ggggacaagtttgtacaaaaaagcaggcttaATGGAGTTGAATGAACATGGTTTCT
pDONR222-RNAi-*MdbHLH093*		R	ggggaccactttgtacaagaaagctgggtAAATCATGGATGCAGGAAGAAATTG
pGBKT7-*MdbHLH093-EcoRI*		F	atggccatggaggccgaattcATGGAGTTGAATGAACATGGTTTCT
pGBKT7-*MdbHLH093-BamHI*		R	ccgctgcaggtcgacggatccTTAGAAGCATCTTCCTCCATATCCT
pGBKT7-*MdbHLH093*$^{\triangle 282\text{-}364}$-*BamHI*		R	ccgctgcaggtcgacggatccAAACTTAGGTGAGTTCCTGACTAGC
pGBKT7-*MdbHLH093*$^{\triangle 248\text{-}364}$-*BamHI*		R	ccgctgcaggtcgacggatccATTGTTGATTCTCTCTAGGAGCTCC
pGBKT7-*MdbHLH093*$^{\triangle 198\text{-}364}$-*BamHI*		R	ccgctgcaggtcgacggatccATTCTTTGATGGCTGCCCCTGCAAC
pGBKT7-*MdbHLH093*$^{\triangle 1\text{-}146}$-*EcoRI*		F	atggccatggaggccgaattcATGCACAACTTGGAGACTCAAGCTG
pGBKT7-*MdbHLH093*$^{\triangle 1\text{-}197}$-*EcoRI*		F	atggccatggaggccgaattcATGCACAACTTGGAGACTCAAGCTG
pSPYNE-*MdbHLH093-BamHI*		F	ggcgcgccactagtggatccATGGAGTTGAATGAACATGGTTTCT
pSPYNE-*MdbHLH093-KpnI*		R	catcccgggagcggtaccGAAGCATCTTCCTCCATATCCTGCA
pDONR222-*MdbHLH093*		F	ggggacaagtttgtacaaaaaagcaggcttaATGGAGTTGAATGAACATGGTTTCT
pDONR222-*MdbHLH093*		R	ggggaccactttgtacaagaaagctgggttGAAGCATCTTCCTCCATATCCTGCA
qRT-*MdbHLH093*		F	TGGTAGCTGGGGAAATTTTG
		R	CTTGATTGACGGTGGTGTTA
qRT-*AtActin2*	AT3G18780	F	TCAATCCAGGAGATGTTTAGG
		R	ACTGCTGGTACTCTGCGACA
qRT-*AtPR2*	AT3G57260	F	GCGTAGTCTAGATGGATGTT
		R	CGCTTGTCCTGCTAGAGGTT
qRT-*AtPR5*	AT1G75040	F	GCACAGAGACACACACAAAA
		R	TGTTCCTTAGAGTGAAGTCTG
qRT-*AtEDS1*	AT3G48090	F	TCATACGCAATCCAAATGTTTAC
		R	AAAAACCTCTCTTGCTCGATCAC
qRT-*AtICS1*	AT1G74710	F	CTTCCGTGACCTTGATCCTTTCT
		R	CAGCGATCTTGCCATTAGGATC
qRT-*MdTubulin*		F	AGGATGCTACAGCCGATGAG
		R	GCCGAAGAACTGACGAGAATC
qRT-*MdPR2*	MD12G1002300	F	F: TGGGACTCGATACCCTAGCCTCT
		R	GCTTGATCACCACCTTCAGAAGGC
qRT-*MdPR5*	MD09G1256000	F	AACTAGCATCCAAAGCTAGCC
		R	CCACAGTCTGCAGTTTCACAAG
qRT-*MdEDS1*	MD14G1188700	F	TGGAGAAAGTGATTTTGGAGAAGC
		R	AGAACCAGATTGTGACAAACGC
qRT-*MdICS1*	MD06G1188700	F	AACATCCCGTGCAAGACGTA
		R	GGCGGCTGAAGACAAAACTG
pGADT7-*MdMYB116-EcoRI*	MD14G1227500	F	gccatggaggccagtgaattcATGTCGACTAAGACTAAAACCCTAA
pGADT7-*MdMYB116-BamHI*		R	cagctcgagctcgatggatccCATTTCGTCCGTGTTCCATATGCTA
pSPYCE-*MdMYB116-BamHI*		F	ggcgcgccactagtggatccATGTCGACTAAGACTAAAACCCTAA

Name	Accession number		Forward (F) and Reverse (R) primer sequence (5’-3’)
pSPYCE-*MdMYB116-KpnI*		R	catcccgggagcggtaccCATTTCGTCCGTGTTCCATATGCTA
pDONR222-*MdMYB116*		F	ggggacaagtttgtacaaaaaagcaggcttaATGTCGACTAAGACTAAAACCCTAA
pDONR222-*MdMYB116*		R	ggggaccactttgtacaagaaagctgggttCATTTCGTCCGTGTTCCATATGCTA
